# Supplementary material for: Fast growth and high-titer bioproduction from renewable formate via metal-dependent formate dehydrogenase in Escherichia coli
Source: Nat Commun. 2025 Jul 1;16:5908. doi: 10.1038/s41467-025-61001-y (PMC12218308; doi:10.1038/s41467-025-61001-y)
Supplement: Supplementary file 1 — Supplementary Information [file 41467_2025_61001_MOESM1_ESM.pdf]

Fast growth and high-titer bioproduction from renewable formate via metal-dependent formate dehydrogenase in *Escherichia coli*

Cowan *et al.*

Supplementary Table 1. Strains used in this study.

Strain name	Genotype	Description	Registry #	Reference
Δ*lpd*	*E. coli* BW25115 Δ*lpd* (F^- DE(*araD-araB*)567 *lacZ4787*(del)::*rrnacB*-3 LAM^- *rph*-1 DE(*rhaD-rhaB*)568 *hsdR*514)	"Energy auxotrophic" strain growth coupled to the rate of NADH regeneration in acetate media	JBx_266836	[1]
Δ*lpd*_cnFDH	Δ*lpd* **pBbS1k_cnFDH**	Energy auxotroph complemented with **pBbS1k_cnFDH**	JBx_266837	This study
Δ*lpd*_psFDH	Δ*lpd* **pBbS1k_psFDH**	Energy auxotroph complemented with **pBbS1k_psFDH**	JBx_266838	This study
K4e	*E. coli* MG1655 (F^- λ^- *ilv*G^- *rfb*-50 *rph*-1) SS9-gC1M, P_{STRONG}-gC2M, SS7-gC3M, SS10-P_{STRONG}-RBSA-*psfdh* (evolved)	Formatotrophic *E. coli* rGlyP strain with one-, and three-carbon modules as well as *psfdh* integrated into genomic safe spots (SS#) [2]. The two-carbon module (glycine cleavage system) was overexpressed from its native genomic locus by strong constitutive promoter P_{STRONG}.	JBx_266839	[3]
K4e *psfdh*::*cat*	K4e ss10-P_{STRONG}-RBSA-*psfdh*::*cat*	K4e with psFDH expression expression cassette deleted by exchange with chloramphenicol acetyltransferase (*cat*) gene (This strain cannot grow on formate alone.)	JBx_266840	This study
K4M	K4e ss10-P_{STRONG}-RBSA-*psfdh*::*cat* **pBbS1k_cnFDH**	K4e with psFDH knockout complemented by expression of cnFDH genes from **pBbS1k_cnFDH**	JBx_266841	This study
K4M*	K4e ss10-P_{STRONG}-RBSA-*psfdh*::*cat* **pBbS1k_cnFDH***	K4e with psFDH knockout complemented by expression of cnFDH genes from **pBbS1k_cnFDH***	JBx_266842	This study
K4Me1	K4M *rpoC* V90D **pBbS1k_cnFDH***	Evolved clone of K4M which has acquired a valine to aspartic acid mutation (codon: GUU to GAU) at position 90 in *rpoC* as well as a single nucleotide polymorphism (SNP) in the 8th nucleotide of the lac operator of **pBbS1k_cnFDH** used to express cnFDH genes (mutant lacO sequence: TGGAATTATGAGCGGATAACAATT)	JBx_266843	This study
K4Me2	K4M *rpoC* +D86 **pBbS1k_cnFDH***	Evolved clone of K4M which has acquired an insertion of aspartic acid (codon:GAU) at position 86 of *rpoC* as well as a SNP in the 8th nucleotide of the lac operator of **pBbS1k_cnFDH** used to express cnFDH genes	JBx_266844	This study
K4Me3	K4M *rpoC* +D86 **pBbS1k_cnFDH***	Whole genome sequencing revealed this clone to be identical to K4Me2	JBx_266845	This study
K4e_mev	K4e **pBbA5a_mevalonate**	K4e transformed with **pBbA5a_mevalonate**	JBx_266846	This study
K4M*_mev	K4M **pBbA5a_mevalonate**	K4M transformed with **pBbA5a_mevalonate**	JBx_266847	This study
K4Me1_mev	K4Me1 **pBbA5a_mevalonate**	K4Me1 transformed with **pBbA5a_mevalonate**	JBx_266848	This study
K4Me2_mev	K4Me2 **pBbA5a_mevalonate**	K4Me2 transformed with **pBbA5a_mevalonate**	JBx_266849	This study
K4Me2_isop	K4Me2 **pBbA5a_isoprenol**	K4Me2 transformed with **pBbA5a_isoprenol**	JBx_266850	This study
XL1-Blue	*E. coli* XL1-blue (*rec*A1 *end*A1 *gyr*A96 *thi*-1 *hsd*R17 *sup*E44 *rel*A1 lac [F´ *proAB lacI*^{q}*Z*Δ*M15* Tn*10* (Tet r)])	Used for DNA storage and preparation		Commercially available (Agilent)

Plasmid names are bolded.

Supplementary Table 2. Doubling times of the fastest formatotrophs recorded.

Reference	Condition	Pathway	Organism	Temp. (°C)	Yield (gDCW/mol)	Doubling time (hr)
Blackmore *et al.*[4]	Aerobic	Calvin Cycle	*Pseudomonas oxalaticus*	30		3.3
Calvey *et al*.[5]	Aerobic	Calvin Cycle	*Cupriavidus necator* CHC124[†]	30		3.3
Pronk *et al*.[6]	Aerobic	Calvin Cycle	*Thiobacillus ferrooxidans*	30	1.3	3.5
Kelly *et al.*[7]	Aerobic	Calvin Cycle	*Thiobacillus strain* A2	30	4.3	3.5
Grunwald *et al.*[8]	Aerobic	Calvin Cycle	*Cupriavidus necator*	30	4.0	3.9
Friedrich *et al*.[9]	Aerobic	Calvin Cycle	*Cupriavidus necator*	30	2.4	4.0
Friedrich *et al.*[10]	Aerobic	Calvin Cycle	*Alcaligenes eutrophus*	30		4.1
**This study**	**Aerobic**	**Synthetic rGlyP**	***Escherichia coli* K-12 K4Me2**	**37**	**3.3**	**4.4**
Goldberg *et al.*[11]	Aerobic	Serine Cycle	*Pseudomonas* 1	32	6.4*	5.8
Kim *et al.*[12]	Aerobic	Synthetic rGlyP	*Escherichia coli* K-12 K4e2	37	3.3	6.0
Pronk *et al*.[6]	Aerobic	Calvin Cycle	*Xanthobacter autotrophicus*	-	4.5	6.9
Dijkhuizen *et al.*[13]	Aerobic	Calvin Cycle	*Pseudomonas oxalaticus* OX1	28	3.4	6.9
Blackmore *et al.*[4]	Aerobic	Serine Cycle	*Pseudomonas* AM-2	30		7.0
Blackmore *et al.*[4]	Aerobic	Serine Cycle	*Pseudomonas extorquens*	30		7.0
Kim *et al.*[3]	Aerobic	Synthetic rGlyP	*Escherichia coli* K-12 K4e	37	2.3	7.7
Blackmore *et al.*[4]	Aerobic	Serine Cycle	*Pseudomonas* AM-1	30		8.0
Chua *et al.*[14]	Anaerobic	Wood-Ljungdahl	*Methanobacterium formicicum*	43	1.4	8.7
Goldberg *et al.*[11]	Aerobic	Serine Cycle	*Pseudomonas* 13.5	32	6.9*	8.7
Goldberg *et al.*[11]	Aerobic	Serine Cycle	*Pseudomonas* M-27	32	5.0	8.7
Goldberg *et al.*[11]	Aerobic	Serine Cycle	*Pseudomonas rosea*	32	3.3	8.7
Lux *et al.*[15]	Anaerobic	Wood-Ljungdahl	*Clostridium formicoaceticum*	37	1.1	9.0
Blackmore *et al.*[4]	Aerobic	Serine Cycle	*Protaminobacter ruber*	30		10.0
Wenk *et al.*[16]	Aerobic	Serine Threonine Cycle	*Escherichia coli* K-12 STC2evo2	37	1.6	10.0
Dronsella *et al.*[17]	Aerobic	Synthetic rGlyP	*Cupriavidus necator* CRG6	30	3.2	11.0
Wood *et al.*[18]	Aerobic	Serine Cycle	*Methylobacterium thiocyanatum*	30	4.4	11.2
Goldberg *et al.*[11]	Aerobic	Serine Cycle	*Pseudomonas* AM-1	32	4.8	11.6
Claassens *et al.*[19]	Aerobic	Synthetic rGlyP	*Cupriavidus necator* CRG4	30	2.6	12.0
Schauer *et al.*[20]	Anaerobic	Wood-Ljungdahl	*Methanobacterium formicicum*	37	1.17	12.6
Morii *et al.*[21]	Anaerobic	Wood-Ljungdahl	*Methanobrevibacter arboriphilus* A2	37	0.8	13.9
Pronk *et al.*[6]	Aerobic	Calvin Cycle	*Thiobacillus versutus*	30	3.0	13.9
Pronk *et al.*[22]	Aerobic	Calvin Cycle	*Thiobacillus acidophilus*	30	2.5	13.9

Synthetic pathways are underlined and this study is **bolded**. [†]adaptively evolved natural formatotroph
*We notice that the formatotrophic yields measured by the natural serine cycle reported so far in only two papers are above 6 gCDW/mole, which is unrealistic, given that theoretical maximum yields for this pathway are not predicted to exceed 5 gCDW/mol [23].

Supplementary Table 3. Product titers of bioproduction from formate.

Reference	Organism/ Strain	Product	Maximum biomass concentration (g/L)	Product titer (g/L)
**This study**	***E. coli* K4Me2**	**Mevalonate**	**4.4***	**3.8**
Cho *et al.* [24]	*Methylobacterium chloromethanicum*	(poly-β-hydroxybutyrate) PHB	~5	1.72
Li *et al.* [25]	*Cupriavidus necator*	Isobutanol and 3-methyl-1-butanol (3 MB)	~1.5*	~0.846 g/L isobutanol and ~0.570 g/L 3 MB
Collas *et al.* [26]	*C. necator*	Crotonate	2.83*	0.148
Kim *et al.* [12]	*E. coli* K4e2	Lactate	~0.56*	0.107
Stöckl *et al.* [27]	*C. necator*	PHB	0.193	0.056–0.073
Janasch *et al.* [28]	*C. necator*	PHB	0.040	0.031
Al Rowaihi *et al.* [29]	*C. necator*	PHB	<0.363*	0.013–0.025
**This study**	***E. coli* K4Me2**	**Isoprenol**	**0.117***	**0.0119**
Jahn *et al.* [30]	*C. necator*	PHB	0.077	0.005
Hegner *et al.* [31]	*Methylorubrum extorquens*	Mesaconate and 2S-methylsuccinate	~0.05*	0.0009 g/L mesaconate and 0.001 g/L μM of 2S-methylsuccinate
Fedorova *et al.* [32]	*E. coli* K4e	PHB	Not reported	Not quantified (only detection)

This study is bolded. *The CDW value (expressed in g/L) was extrapolated using a CDW/OD_{600} ratio of 0.36.[8]
This table was adapted and updated from Collas *et al.* [26]**.**

Supplementary Table 4. Primers used in this study.

Primer	Sequence
**AC1**	agcatagagatctgctgccgatctgcgcaatg
**AC2**	catagaccaattgctactccagcatcgcccga
**AC3**	cataatccaattgtcactccaccggtgcttaa
**AC4**	gatttcgaactcgtgaccg
**AC5**	agactaagaattcaaaagattaggatccaaactcgagta
**AC6**	atgtatatctccttcttaaagatcctttagatccagatcc
**AC7**	agaaggagatatacatatgaccgtttacaca
**AC8**	tcttttgaattcttagtctactttcagaccct
**AC9**	accgtcacccagtcagcgttggcttcgaagcacatacgcgaaaggattttggcaaagcctcgcaatc
**AC10**	atgacaacgatccggtggatatcctcatcaccatggcggcggtcgatgcccctgggccaacttttggcgaaaatg

Supplementary Table 5. Plasmids used in this study.

Plasmid	Description	Registry #	Reference
**pBbS1k_cnFDH**	Expression construct for *Cupriavidus necator* FDH (cnFDH) containing genes *fdsABCDG* under control of the trc promoter	JBx_266054	This study
**pBbS1k_psFDH**	Expression construct for *Pseudomonas* sp. 101 FDH (psFDH) under control of the trc promoter	JBx_266055	This study
**pBbS1k_cnFDH***	Expression construct for cnFDH genes with mutation in lac operator	JBx_266056	This study
**pBbA5a_mevalonate**	Expression construct for *Escherichia coli* atoB, *Saccharomyces cerevisiae* HMGS and *Delftia acidovorans* HMGR under control of the lacUV5 promoter	JBx_266057	This study
**pBbA51a_isoprenol**	Expression construct for *E. coli* atoB, *S. cerevisiae* HMGS and *D. acidovorans* HMGR under control of the lacUV5 promoter as well as *S. cerevisiae* PMD with R74G mutation and *S. cerevisiae* mevaloante kinase (MK) under control of the trc promoter	JBx_266058	This study

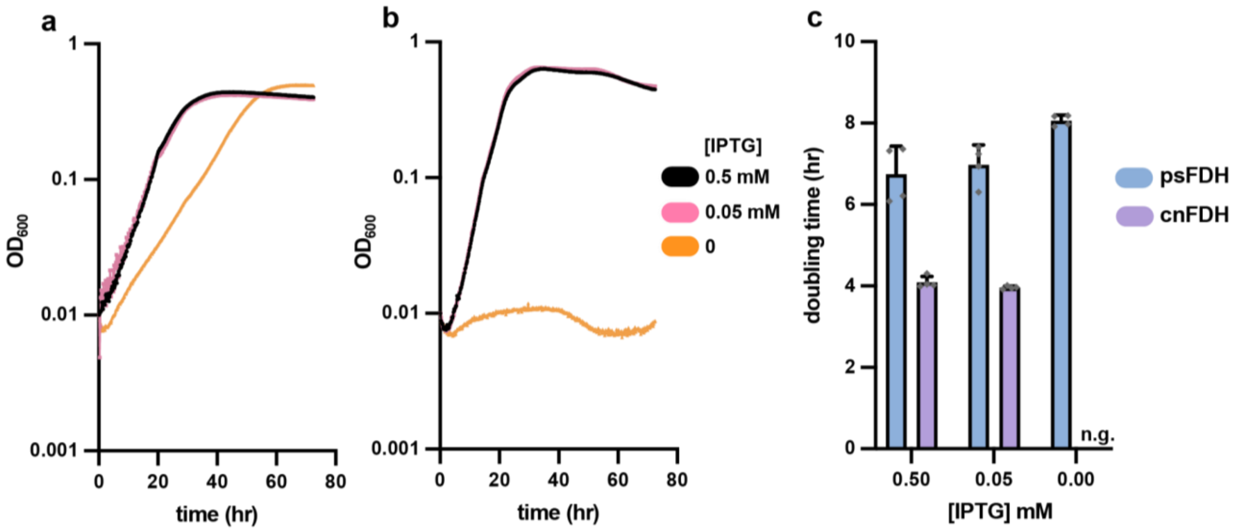

Supplementary Figure 1. Optimization of FDH induction in Bw25113 Δ*lpd*. a, Growth of Δ*lpd* strain containing pBb_S1k_psFDH cultivated in minimal medium containing 20mM acetate and 60mM formate. b, Growth of strain Δ*lpd*_cnFDH cultivated in minimal medium containing 20mM acetate and 60mM formate. c, corresponding doubling times for the growth curves shown in panels a and b. Doubling time is minimized for Δ*lpd* expressing psFDH at 0.5mM IPTG and at 0.05mM IPTG for Δ*lpd*_cnFDH (n.g. is no growth). Source data are provided as a Source Data file.

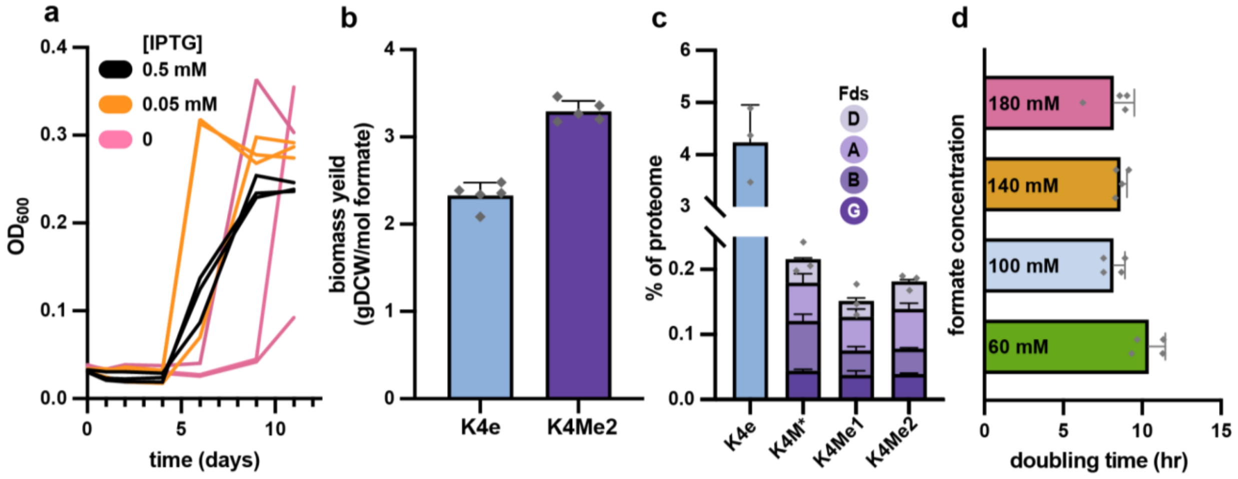

Supplementary Figure 2. Supporting information for the replacement of the energy module of K4e. a, Initial growth of strain K4M before adaptive evolution. Growth is characterized by a long lag phase and inconsistent lag phase length between replicates (therefore replicates were not averaged and are displayed individually). Even with only leaky expression of cnFDH the K4M strain is able to grow albeit with a long and inconsistent lag phase. b, Biomass yield of K4e and K4Me2 strains showing significantly greater biomass yields in K4Me2 (3.3 ± 0.1) relative to K4e (2.3 ± 0.1) (p=3E-6) (n=5). c, Proteomic analysis of FDH expression in K4e and K4M-derived strains reveals cnFDH expression consistent with that observed in Δlpd. However, the optimized expression of psFDH in K4e is significantly (p=0.006) lower than the expression level which allowed for the minimal doubling time in Δlpd (n=3). d, Formate titration in K4e shows a doubling time minimum at 100 mM formate. This concentration was then used in subsequent experiments. Source data are provided as a Source Data file.

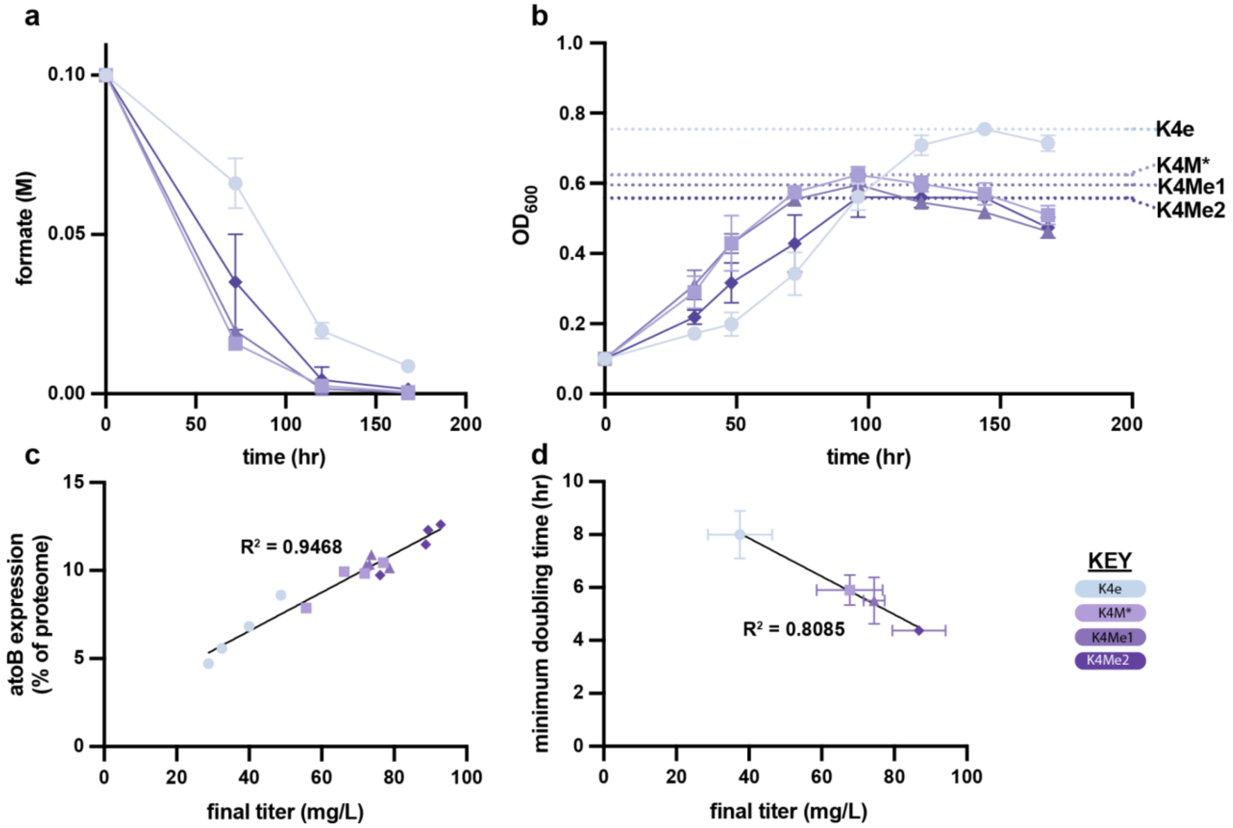

Supplementary Figure 3. Formate consumption and growth during mixotrophic bioproduction of mevalonate. a, Formate consumption during bioproduction of mevalonate from 100mM formate and 1mM glucose. Formate was consumed more rapidly and completely in the K4M-derived strains. b, Growth of K4e and K4M-derived strains during mixotrophic bioproduction from 100mM formate and 1mM glucose. All K4M-derived strains grew to a significantly lower final OD than K4e (K4M*_mev p=0.0001, K4Me1_mev p=0.0001, K4Me2_mev p=0.0007) (n=4). Given that overall formate consumption is similar and the titers of mevalonate achieved for K4M-derived strains was significantly higher than the K4e strain, this indicates a redirection of carbon flux away from central metabolism and into product formation, a desirable characteristic for an industrial strain. c. The final titer of each strain was highly correlated (R^2=0.9468) with the expression of AtoB, the first enzyme in the mevalonate pathway, responsible for diverting flux from central metabolism into product formation. d. The final titer of each strain was also highly correlated (R^2=0.8085) with the minimal doubling time of each strain. Source data are provided as a Source Data file.

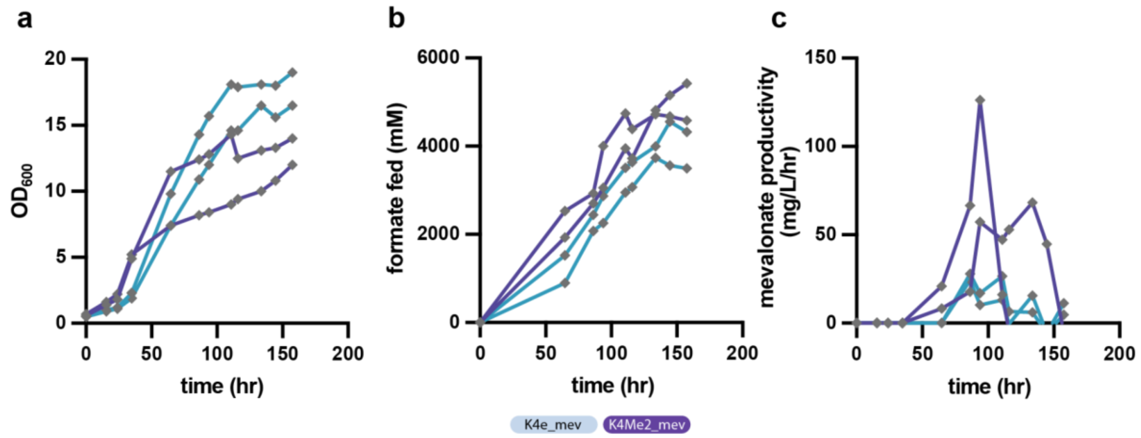

Supplementary Figure 4. Growth and formate consumption during formatotrophic fed-batch bioproduction of mevalonate. a, Optical density at 600nm of bioreactors during formatotrophic bioproduction of mevalonate. Induction with 0.5 mM IPTG occurred at 65 hours post inoculation. K4Me2 strain reached a lower final cell density, consistent with results at small scale. b, Formate fed during fed-batch, formatotrophic bioproduction of mevalonate. c, Productivity during fed-batch, formatotrophic bioproduction of mevalonate. Source data are provided as a Source Data file.

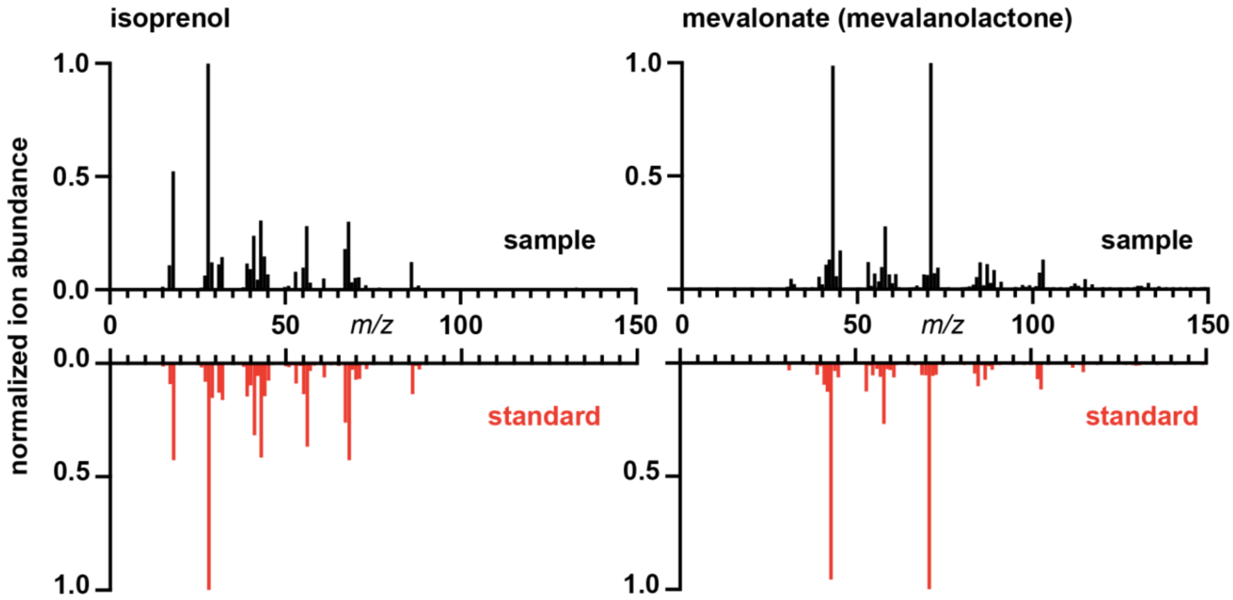

Supplementary Figure 5. Mass spectra of isoprenol and derivatized mevalonate. To confirm the authenticity of formate derived isoprenol and mevalonate, GCMS was conducted to obtain mass spectra for comparison with an authentic standard. Mevalonate is too polar for compatibility with GCMS and was therefore derivatized to mevalonolactone before analysis. Both isoprenol and mevalonolactone mass spectra were identified and are shown here alongside their authentic standards. Source data are provided as a Source Data file.

Supplementary references

1. Wenk, S. *et al.* An 'energy-auxotroph' *Escherichia coli* provides an *in vivo* platform for assessing NADH regeneration systems. *Biotechnol. Bioeng.* **117**, 3422–3434 (2020).
2. Bassalo, M. C. *et al.* Rapid and efficient one-step metabolic pathway integration in *E. coli*. *ACS Synth. Biol.* **5**, 561–568 (2016).
3. Kim, S. *et al.* Growth of *E. coli* on formate and methanol via the reductive glycine pathway. *Nat. Chem. Biol.* **16**, 538–545 (2020).
4. Blackmore, M. A. & Quayle, J. R. Microbial growth on oxalate by a route not involving glyoxylate carboligase. *Biochem. J.* **118**, 53–59 (1970).
5. Calvey, C. H. *et al.* Improving growth of *Cupriavidus necator* H16 on formate using adaptive laboratory evolution-informed engineering. *Metab. Eng.* **75**, 78–90 (2023).
6. Pronk, J. T. *et al.* Growth of *Thiobacillus ferrooxidans* on formic acid. *Appl. Environ. Microbiol.* **57**, 2057–2062 (1991).
7. Kelly, D. P., Wood, A. P., Gottschal, J. C. & Kuenen, J. G. Autotrophic metabolism of formate by *Thiobacillus* strain A2. *J. Gen. Microbiol.* **114**, 1–13 (1979).
8. Grunwald, S. *et al.* Kinetic and stoichiometric characterization of organoautotrophic growth of *Ralstonia eutropha* on formic acid in fed-batch and continuous cultures: Organoautotrophic growth of *Ralstonia eutropha*. *Microb. Biotechnol.* **8**, 155–163 (2015).
9. Friedrich, C. G., Bowien, B. & Friedrich, B. Formate and oxalate metabolism in *Alcaligenes eutrophus*. *J. Gen. Microbiol.* **115**, 185–192 (1979).
10. Friedrich, C. G., Friedrich, B. & Bowien, B. Formation of enzymes of autotrophic metabolism during heterotrophic growth of *Alcaligenes eutrophus*. *Microbiology* **122**, 69–78 (1981).
11. Goldberg, I., Rock, J. S., Ben-Bassat, A. & Mateles, R. I. Bacterial yields on methanol, methylamine, formaldehyde, and formate. *Biotechnol. Bioeng.* **18**, 1657–1668 (1976).
12. Kim, S. *et al.* Optimizing *E. coli* as a formatotrophic platform for bioproduction via the reductive glycine pathway. *Front. Bioeng. Biotechnol.* **11**, 1091899 (2023).
13. Dijkhuizen, L., Wiersma, M. & Harder, W. Energy production and growth of *Pseudomonas oxalaticus* OX1 on oxalate and formate. *Archives of Microbiology* **115**, 229–236 (1977).
14. Chua, H. B. & Robinson, J. P. Formate-limited growth of *Methanobacterium formicium* in steady-state cultures. *Arch. Microbiol.* **135**, 158–160 (1983).
15. Lux, M. F. & Drake, H. L. Re-examination of the metabolic potentials of the acetogens *Clostridium aceticum* and *Clostridium formicoaceticum*: Chemolithoautotrophic and aromatic-dependent growth. *FEMS Microbiol. Lett.* **95**, 49–56 (1992).
16. Wenk, S. *et al.* Evolution-assisted engineering of *E. coli* enables growth on formic acid at ambient CO_2 via the Serine Threonine Cycle. *Metab. Eng.* **88**, 14–24 (2024).
17. Dronsella, B. *et al.* Engineered synthetic one-carbon fixation exceeds yield of the Calvin cycle. *bioRxiv* (2022) doi:10.1101/2022.10.19.512895.
18. Wood, A. P. *et al.* A novel pink-pigmented facultative methylotroph, *Methylobacterium thiocyanatum* sp. nov., capable of growth on thiocyanate or cyanate as sole nitrogen sources. *Arch. Microbiol.* **169**, 148–158 (1998).
19. Claassens, N. J. *et al.* Replacing the Calvin cycle with the reductive glycine pathway in *Cupriavidus necator*. *Metab. Eng.* **62**, 30–41 (2020).
20. Schauer, N. L. & Ferry, J. G. Metabolism of formate in *Methanobacterium formicicum*. *J. Bacteriol.* **142**, 800–807 (1980).
21. Morii, H., Nishihara, M. & Koga, Y. Isolation, characterization and physiology of a new formate-assimilable methanogenic strain (A2) of *Methanobrevibacter arboriphilus*. *Agric. Biol. Chem.* **47**, 2781–2789 (1983).
22. Pronk, J. T., de Bruijn, P., van Dijken, J. P., Bos, P. & Kuenen, J. G. Energetics of mixotrophic and autotrophic C1-metabolism by *Thiobacillus acidophilus*. *Arch. Microbiol.* **154**, 576–583 (1990).
23. Cotton, C. A., Claassens, N. J., Benito-Vaquerizo, S. & Bar-Even, A. Renewable methanol and formate as microbial feedstocks. *Curr. Opin. Biotechnol.* **62**, 168–180 (2020).
24. Cho, D. H., Jang, M. G. & Kim, Y. H. Formatotrophic production of poly-β-hydroxybutyric acid (PHB) from *Methylobacterium* sp. Using formate as the sole carbon and energy source. *Hwahak Konghak* **54**, 719–721 (2016).
25. Li, H. *et al.* Integrated electromicrobial conversion of CO_2 to higher alcohols. *Science* **335**, 1596 (2012).
26. Collas, F. *et al.* Engineering the biological conversion of formate into crotonate in *Cupriavidus necator*. *Metab. Eng.* **79**, 49–65 (2023).

27. Stöckl, M., Harms, S., Dinges, I., Dimitrova, S. & Holtmann, D. From CO_2 to bioplastic - coupling the electrochemical CO_2 reduction with a microbial product generation by drop-in electrolysis. *ChemSusChem* **13**, 4086–4093 (2020).
28. Janasch, M. *et al.* Thermodynamic limitations of PHB production from formate and fructose in *Cupriavidus necator*. *Metab. Eng.* **73**, 256–269 (2022).
29. Al Rowaihi, I. S. *et al.* Poly(3-hydroxybutyrate) production in an integrated electromicrobial setup: Investigation under stress-inducing conditions. *PLoS One* **13**, e0196079 (2018).
30. Jahn, M. *et al.* Protein allocation and utilization in the versatile chemolithoautotroph *Cupriavidus necator*. *eLife* **10**, (2021).
31. Hegner, R., Neubert, K., Kroner, C., Holtmann, D. & Harnisch, F. Coupled electrochemical and microbial catalysis for the production of polymer bricks. *ChemSusChem* **13**, 5295–5300 (2020).
32. Fedorova, D. *et al.* Demonstration of bioplastic production from CO_2 and formate using the reductive glycine pathway in *E. coli*. *bioRxiv* (2023) doi:10.1101/2023.12.02.569694.
